# Integrative Analysis of DNA Methylation and Gene Expression Profiles Identifies Colorectal Cancer-Related Diagnostic Biomarkers

**DOI:** 10.3389/pore.2021.1609784

**Published:** 2021-07-21

**Authors:** Mingyue Xu, Lijun Yuan, Yan Wang, Shuo Chen, Lin Zhang, Xipeng Zhang

**Affiliations:** ^1^ Department of Colorectal Surgery, Tianjin Union Medical Center, Tianjin, China; ^2^ Department of Traditional Chinese Medicine, Shanghai Pudong New Area People’s Hospital, Shanghai, China

**Keywords:** Colorectal cancer, DNA methylation, logistic regression model, CpG island methylator phenotype, The Cancer Genome Atlas, Gene Expression Omnibus, diagnosis

## Abstract

**Background:** Colorectal cancer (CRC) is a common human malignancy worldwide. The prognosis of patients is largely frustrated by delayed diagnosis or misdiagnosis. DNA methylation alterations have been previously proved to be involved in CRC carcinogenesis.

**Methods:** In this study, we proposed to identify CRC-related diagnostic biomarkers by analyzing DNA methylation and gene expression profiles. TCGA-COAD datasets downloaded from the Cancer Genome Atlas (TCGA) were used as the training set to screen differential expression genes (DEGs) and methylation CpG sites (dmCpGs) in CRC samples. A logistic regression model was constructed based on hyper-methylated CpG sites which were located in downregulated genes for CRC diagnosis. Another two independent datasets from the Gene Expression Omnibus (GEO) were used as a testing set to evaluate the performance of the model in CRC diagnosis.

**Results:** We found that CpG island methylator phenotype (CIMP) was a potential signature of poor prognosis by dividing CRC samples into CIMP and noCIMP groups based on a set of CpG sites with methylation standard deviation (sd) > 0.2 among CRC samples and low methylation levels (mean β < 0.05) in adjacent samples. Hyper-methylated CpGs tended to be more closed to CpG island (CGI) and transcription start site (TSS) relative to hypo-methylated CpGs (*p*-value < 0.05, Fisher exact test). A logistic regression model was finally constructed based on two hyper-methylated CpGs, which had an area under receiver operating characteristic curve of 0.98 in the training set, and 0.85 and 0.95 in the two independent testing sets.

**Conclusions:** In conclusion, our study identified promising DNA methylation biomarkers for CRC diagnosis.

## Introduction

Colorectal cancer (CRC) is a frequently lethal disease with high incidence. In most cases, CRC usually starts as a polyp (a noncancerous growth that develops in the lining of the colon and rectum), and does not have obvious symptoms until it becomes difficult to cure [1]. Therefore, CRC can largely be prevented by the early detection and removal of precursor lesions [2]. There are two main types of CRC screening strategies: stool tests (such as fecal occult blood testing) and structural exams, including colonoscopy, double-contrast barium enema, and computed tomographic colonography, etc. [3]. However, the effects of these tests are not satisfactory in clinical applications because of their complex protocols and limited sensitivity and specificity [4]. Since the occurrence of CRC is driven by the accumulation of genetic abnormalities, there has been an increasing number of gene abnormality-based technologies available for CRC screening in the last decade [5]. Even though several tests have already been used in clinical practice, current options still do not have enough sensitivity and specificity to serve as general screening [6].

The results of CRC epigenome assessment reveal that almost all CRCs have aberrantly methylated genes, which play pathological roles in CRC development [7]. Meanwhile, the heterogeneity of methylation characteristics among individuals is closely associated with the prognosis [8]. There are two types of methylation associated with CRC progression: age-related methylation (type A), and cancer-specific methylation (type C) [9]. Among the type C methylation, DNA hypermethylation of CpG-rich promoters, which result in switching off tumor suppressor genes, has been recognized as a subgroup of CRCs. CpG island methylator phenotype (CIMP) defines the overall methylation-mediated gene expression pattern in a sample by the methylation status of specific gene promoters [10]. Some studies proposed CIMP status as the most promising predictor of all CRC biomarker candidates [11], however, this view is still controversial and needs more research to provide rigorous evidence.

In this study, we performed an integrated analysis of DNA methylation and gene expression profiles of CRC. The data downloaded from The Cancer Genome Atlas (TCGA) were used as a training set, and that from Gene Expression Omnibus (GEO) datasets were used as a testing set. The differential expression genes (DEGs) and methylation CpG sites (dmCpGs) in CRC samples were identified, and a logistic regression model was constructed based on the hypermethylated CpG sites which were located in downregulated genes for CRC diagnosis. Finally, the prediction accuracy of the constructed model was evaluated. We believe that these results can contribute to research on the screening of early diagnostic markers for CRC.

## Materials and Methods

### Datasets

DNA methylation and gene expression profiles of the TCGA-COAD dataset which contained 407 CRC and 46 adjacent samples were downloaded from TCGA and used as a training set. The testing set consisted of two independent DNA methylation datasets, including GSE79740 [12] which contained 44 CRC samples and 10 normal samples, and GSE42752 [13] which contained 22 CRC and 41 normal samples from GEO.

### Definition of CpG Island Methylator Phenotype

CpG island methylator phenotype (CIMP) which defines the overall methylation-mediated gene expression pattern in a sample by the methylation status of specific gene promoters and heterogeneity of methylation characteristics among individuals and is closely associated with tumorigenesis and prognosis was first proposed in CRC [13]. In this study, we classified CRC samples into CIMP or noCIMP groups through a k-means clustering method based on the methylation levels of CpG sites with methylation sd > 0.2 among CRC samples and mean methylation levels <0.05 in adjacent samples. Optimal clustering number was determined by the within-cluster sum of squares (wss) method.

## Differential DNA Methylation and Gene Expression Analysis

DNA methylation and gene expression profiles were first processed, including the removal of CpG sites and genes with missing values in more than 10% of samples, and then the remaining missing values were added through the R Bioconductor impute package (https://bioconductor.org/packages/release/bioc/html/impute.html). We used paired t-test to screen differential methylation CpG sites (DMCs) between the 44 pairs of CRC and adjacent samples with the thresholds of absolute β (methylation level) difference >0.2 and FDR adjusted *p*-value < 0.05. Differential expression genes (DEGs) between paired CRC and adjacent samples were measured through the *edgeR* Bioconductor package [14] based on the raw count data. Genes with absolute log2 (fold change) > 1 and FDR adjusted *p*-value < 0.05 were determined as differentially expressed.

## Construction of CRC Diagnostic Model

To screen reliable CRC diagnostic biomarkers, we selected hyper-methylated DMCs in CRC samples and filtered out those not in promoters and with β > 0.05 in adjacent samples; the remaining DMCs are hereafter referred to as ProHyperDMCs. Hyper-methylation in promoters were usually associated with repressed gene expression, so we further selected CpGs from ProHyperDMCs that were located in downregulated genes in CRC samples as promising CRC diagnostic biomarkers. A logistic regression model was finally constructed using sample type, i.e., CRC or normal, as response variables and CpGs’ β values were used as predict variables in the training set.

### Evaluation of the CRC Diagnostic Model

The sample types of CRC and normal samples in GSE79740 and GSE42752 as testing sets were predicted through the CRC diagnostic model. Receiver operating characteristic curve (ROC) was plotted and area under curve (AUC) was calculated by using *pROC* [15] and *ROCR* [16] Bioconductor packages for evaluating the CRC diagnostic model's performance.

## Results

### CIMP Was Associated With Poor CRC Overall Survival

**FIGURE 1:**
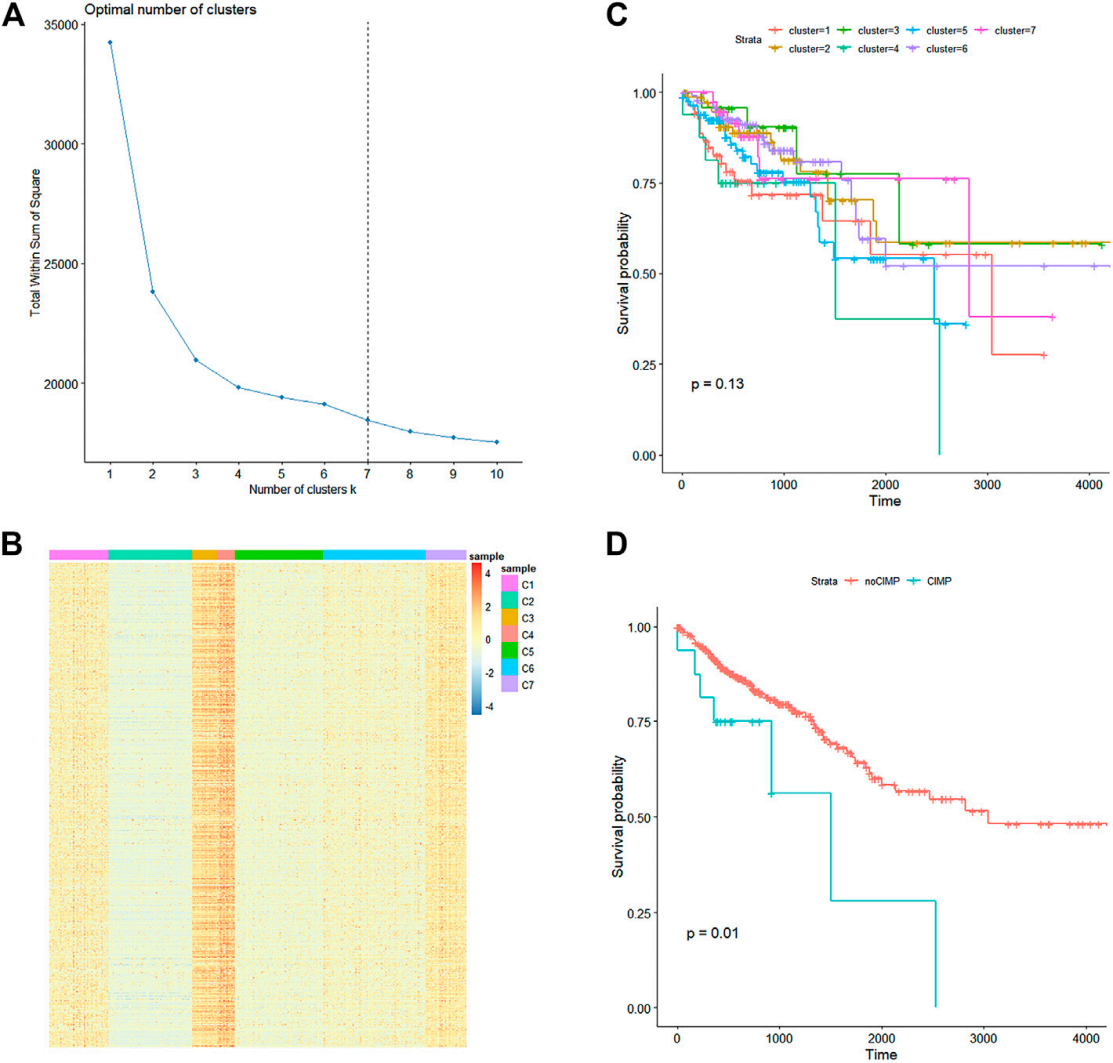
Methylation landscape of 407 CRC samples in the TCGA-COAD dataset. **(A)** Line graph of total within sum of square (Y-axis) vs. cluster number (X-axis) obtained through the within-cluster sum of squares method which defined the optimal cluster number as 7. **(B)** K-means cluster analysis of the 407 CRC samples based on their Euclidean distance calculated through β values of the 3,561 CpG sites with the cluster number specified as 7. **(C)** Kaplan-Meier curves of CRC samples stratified by their cluster. **(D)** Kaplan-Meier curves of CRC samples in cluster 4 (CIMP group) and other clusters (noCIMP). Abbreviations: CRC, colorectal cancer; CRMP, CpG island methylator phenotype (CIMP).

A total of 3,561 CpGs were obtained, which satisfied the condition that the sd of β values was smaller than 0.2 among the 407 CRC samples and mean β values of the 46 adjacent samples were smaller than 0.05 in the training set. K-means clustering was applied to the 406 samples based on their Euclidean distance calculated through the β values of the 3,561 CpGs. Seven was considered as the optimal cluster number by the wss method for the gentler incline from this point as shown in Figure 1A. Cluster four had significantly higher overall methylation levels across almost all of the 3,561 CpGs (Figure 1B) than those of other clusters and thus samples in this cluster were considered to have CIMP. Then cluster 4 with CIMP was compared with other clusters, our results showed that there was a significant difference between cluster four and cluster 2, cluster 3, cluster 6, and cluster 7 (Supplementary Figure 1). To explore the relation between CIMP and CRC patients' prognosis, we estimated the overall survival (OS) of CRC samples using the Kaplan-Meier method and determined the significance of OS differences among the seven CRC clusters through the log-rank test. As a result, the *p*-value was determined as 0.13 compared to CRC samples' OS in the seven clusters although cluster four had a relative lower survival probability than that of other clusters (Figure 1C). We then combined CRC samples in all clusters except for cluster four and defined them as noCIMP, and tested the OS differences between the two CRC groups. Strikingly, the survival probability of CRC patients in cluster 4, i.e., CIMP group, was significantly lower than that in the noCIMP group (log-rank *p*-value = 0.01, Figure 1D).

### Differential Methylation CpGs

**FIGURE 2:**
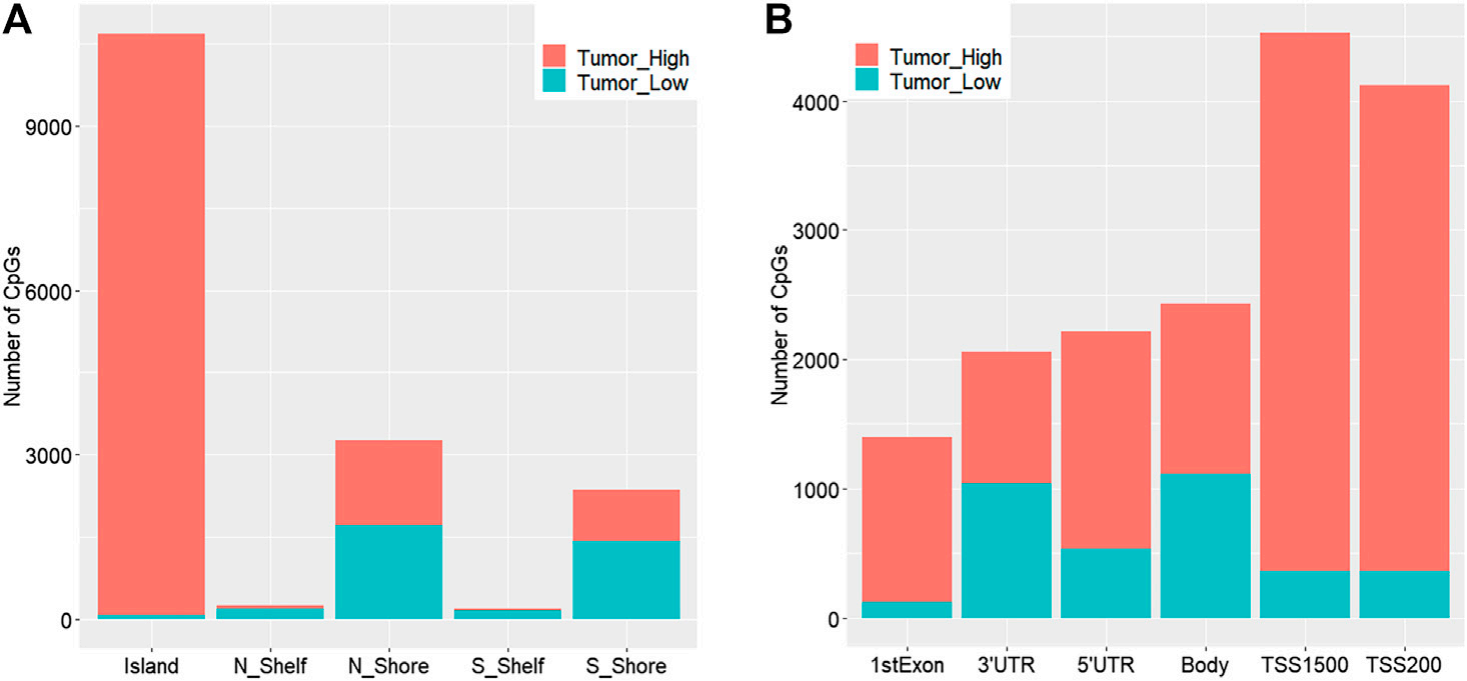
Distribution of genomic regions of differential methylated CpG sites according to distance relative to CpG islands **(A)** and transcription start sites (TSSs) **(B)**. N_Shore and S_Shore are 200-bp upstream and downstream of a CpG island, and N_Shelf and S_Shelf are 200–1500-bp upstream and downstream of a CpG island.

We obtained a total of 16,747 dmCpGs in CRC samples compared to 3,534 hypo- and 13,213 hyper-methylated sites in adjacent samples. The distribution of hyper- and hypo-methylated CpGs across genomic regions relative to CpG islands and transcription start sites (TSSs) are provided in Figure 2A and Figure 2B, respectively. Hyper-methylated CpGs significantly tended to be located in CpG islands and promoters (i.e., TSS200 and TSS1500 in Figure 2B), compared with hypo-methylated CpGs with global distribution across the whole genome (chi-square test, *p* = 0.037).

### CRC Diagnostic Biomarkers

**FIGURE 3:**
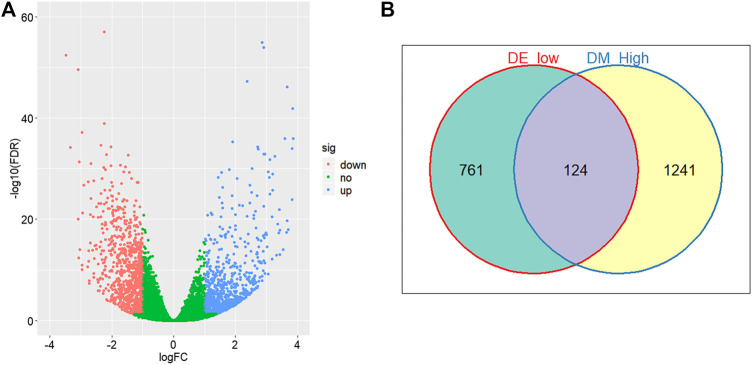
Differential methylation and expression analysis. **(A)** Volcano plot of logarithmic-transformed FDR (Y-axis) vs. logarithmic-transformed fold change of gene expression values (X-axis). Red, blue, and green dots represent downregulated, upregulated, and non-differential expression genes in CRC samples, respectively. **(B)** Venn diagram of hyper-methylation and downregulated expression genes in CRC samples. Hyper-methylation genes are defined as genes containing at least one hyper-methylation CpG site.

**FIGURE 4:**
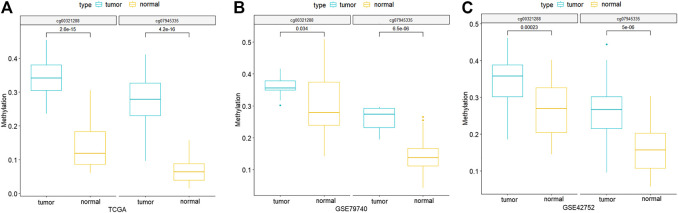
Methylation differences of the two CpG signatures between normal and CRC samples of TCGA-COAD **(A)**, GSE79740 **(B)**, and GSE42752 **(C)** datasets. *p*-values are calculated by Wilcoxon rank sum test.

Through comparing the gene expression profiles between CRC and adjacent samples, we obtained 885 down- and 1,000 upregulated genes in CRC samples as shown in Figure 3A. Cross-analysis of 1,365 genes annotated by the 13,213 hyper-methylated CpGs and the 885 downregulated DEGs identified a total of 124 overlaps (Figure 3B) which covered 195 hyper-methylated CpGs. The detailed results of DEGs are shown in Supplementary Table 1. Then after removing the sites that were not on promoters or sex chromosomes, 25 CpGs remained. Subsequently, the cross-analysis of these 25 CpGs and the hypo-methylated CpGs in normal samples (β value < 0.05) identified two overlap sites. Finally, cg07945335 and cg00321288 among the 195 CpGs located in the promoter of CD300LG and MGAT4C were selected for CRC diagnostic model construction. As shown in Figure 4, those two CpGs were hyper-methylated in CRC samples of the training set and the testing set, which indicated their reliability.

### Construction and Evaluation of CRC Diagnostic Model

**FIGURE 5:**
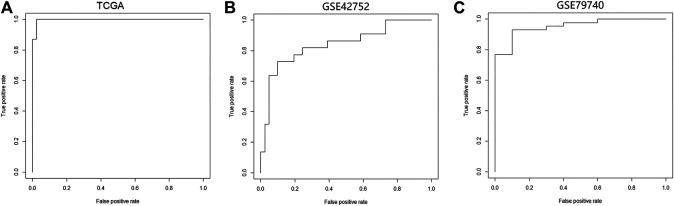
Performance of the logistic regression diagnostic model constructed by the two CpG signatures in CRC. **(A)** ROC curve of the logistic regression model in the TCGA-COAD dataset. **(B)** ROC curve of the logistic regression model in the GSE42752 dataset. **(C)** ROC curve of the logistic regression model in the GSE79740 dataset. Logistic regression diagnostic model's performance in CRC was evaluated by the area under curve (AUC). Abbreviation: ROC, receiver operating characteristic curve.

We constructed a logistic regression model using the sample type and β values of cg07945335 and cg00321288 in the training set as response and predict variables, respectively. AUC of the model could achieve 0.98, 0.85, and 0.95 when applied to the training set, and GSE42752 and GSE79740 of the testing set (Figure 5), which illustrated the good performance of the model in CRC diagnosis.

## Discussion

The vast majority of human cancer cells harbor both genetic and epigenetic abnormalities, which allow them to escape from chemotherapy and host immune surveillance [17]. Hence, a growing number of efforts on the analysis of high-throughput sequencing-based epigenome data, including DNA methylation and histone modifications, has been advanced for the need of individualized therapies [18]. In addition, methylation characteristics were also closely related to the prognosis of CRC patients [19]. For example, UHRF1, FOXE1, AXIN2, and DKK1 have recently been defined as biomarkers that support oncogenic properties, and high expressions of these genes predict reduced CRC patient survival [20–22].

CIMP status was first found in CRC, and this subtype had distinct histological and molecular features [23]. In this study, we first clustered the CRC samples based on methylation of CpG sites, and identified the patients with CIMP. The OS analysis revealed that the CIMP status was significantly associated with the prognosis of CRC patients (Figure 1), which was consistent with the literature report. Furthermore, we performed a cross-analysis between differential methylation sites and differential genes, and identified cg07945335 and cg00321288 as the key genes for CRC diagnostic model construction, which were located in the promoter of CD300LG and MGAT4C, respectively.

CD300LG protein, a member of the CD300 family, is a type I cell surface glycoprotein is that exclusively expressed in the capillary endothelium [24]. CD300LG mediates molecular traffic across the capillary endothelium, responds to the immunological environment, and is implicated in lymphocyte binding and transmigration [24, 25]. Herein, we reported on the important role of CD300LG in the CRC process for the first time, since leukocyte diapedesis through the vasculature involves critical adhesive interactions with endothelial cells, and both leukocytes and cancer cells express similar surface receptors capable of binding endothelial adhesion molecules [26]. Therefore, we speculated that CD300LG probably affected transendothelial migration of CRC cancer cells by regulating the response of cancer cells to the immune microenvironment, which will be confirmed in our future studies. Mannosyl (alpha-1,3-)-glycoprotein beta-1,4-N-acetylglucosaminyltransferase (MGAT4C), is a member of the MGAT4 family [27]. Demichelis F et al. investigated the possible function of MGAT4C in prostate cancer through gene overexpression and knockdown experiments [28]. The results revealed that MGAT4C expression was related to the proliferation and migration of prostate cancer cells. However, the function of this MGAT4C in CRC still needs more exploration.

We constructed a CRC diagnostic model based on cg07945335 and cg00321288, and used GEO data as a validation set to determine the specificity and sensitivity of these two key genes as diagnostic biomarkers, and the results indicated the good performance of the diagnostic model in CRC.

In conclusion, this study identified promising DNA methylation biomarkers for CRC diagnosis through an integrative analysis of DNA methylation and gene expression data. Nevertheless, there are also some limitations in this study. First, the expressions of these biomarkers have not been verified by clinical samples, and the biological function of them is not clear. Second, since the occurrence and development of CRC are related to some high risk factors such as age, the inclusion of other clinical factors and the expansion of the sample size will help to improve the accuracy of the model.

## Data Availability

The datasets analyzed for this study can be found in TCGA-COAD (https://portal.gdc.cancer.gov/), GEO (https://www.ncbi.nlm.nih.gov/geo/query/acc.cgi?acc=GSE79740, https://www.ncbi.nlm.nih.gov/geo/query/acc.cgi?acc=GSE42752).

## References

[1] Bray C Bell LN Liang H Collins D Yale SH. Colorectal Cancer Screening. WMJ (2017) 116:27–33. 29099566

[2] Issa IA Noureddine M. Colorectal Cancer Screening: An Updated Review of the Available Options. Wjg (2017) 23:5086–96. 10.3748/wjg.v23.i28.5086 28811705 PMC5537177

[3] Levin B Lieberman DA Mcfarland B Smith RA Brooks D Andrews KS Screening and Surveillance for the Early Detection of Colorectal Cancer and Adenomatous Polyps, 2008: a Joint Guideline from the American Cancer Society, the US Multi-Society Task Force on Colorectal Cancer, and the American College of Radiology. CA: A Cancer J Clinicians (2008) 58:130–60. 10.3322/ca.2007.0018 18322143

[4] Vatandoost N Ghanbari J Mojaver M Avan A. Ghayour-Mobarhan M. Nedaeinia R. Early Detection of Colorectal Cancer: from Conventional Methods to Novel Biomarkers. J Cancer Res Clin Oncol (2016) 142:3413–51. 10.1007/s00432-015-1928-z PMC11819464 25687380

[5] Lao VV Grady WM. Epigenetics and Colorectal Cancer. Nat Rev Gastroenterol Hepatol (2011) 8:686–700. 10.1038/nrgastro.2011.173 22009203 PMC3391545

[6] Van Lanschot MCJ Bosch LJW De Wit M Carvalho B Meijer GA. Early Detection: the Impact of Genomics. Virchows Arch (2017) 471:165–73. 10.1007/s00428-017-2159-2 28573511

[7] Marmol I Sanchez-de-diego C Pradilla Dieste A Dieste A. P. Cerrada E. Yoldi M. J. R. Colorectal Carcinoma: A General Overview and Future Perspectives in Colorectal Cancer. Int J Mol Sci (2017) 18. 10.3390/ijms18010197 PMC5297828 28106826

[8] Weisenberger DJ Liang G Lenz H-J. DNA Methylation Aberrancies Delineate Clinically Distinct Subsets of Colorectal Cancer and Provide Novel Targets for Epigenetic Therapies. Oncogene (2018) 37:566–77. 10.1038/onc.2017.374 28991233 PMC7491233

[9] Wynter CVA Walsh MD Higuchi T. Methylation Patterns Define Two Types of Hyperplastic Polyp Associated with Colorectal Cancer. Gut (2004) 53:573–80. 10.1136/gut.2003.030841 15016754 PMC1774017

[10] Naumov VA Generozov EV Zaharjevskaya NB Matushkina DS Larin AK Chernyshov SV Genome-scale Analysis of DNA Methylation in Colorectal Cancer Using Infinium HumanMethylation450 BeadChips. Epigenetics (2013) 8:921–34. 10.4161/epi.25577 23867710 PMC3883769

[11] Gündert M Edelmann D Benner A Jansen L Jia M Walter V Genome-wide DNA Methylation Analysis Reveals a Prognostic Classifier for Non-metastatic Colorectal Cancer (ProMCol Classifier). Gut (2019) 68:101–10. 10.1136/gutjnl-2017-314711 29101262

[12] Zhang F Wang L Li Y. Optimizing Mesoderm Progenitor Selection and Three-Dimensional Microniche Culture Allows Highly Efficient Endothelial Differentiation and Ischemic Tissue Repair from Human Pluripotent Stem Cells. Stem Cel Res Ther (2017) 8:6. 10.1186/s13287-016-0455-4 PMC5259899 28114972

[13] Nazemalhosseini Mojarad E Kuppen PJ Aghdaei HA Zali MR. The CpG Island Methylator Phenotype (CIMP) in Colorectal Cancer. Gastroenterol Hepatol Bed Bench (2013) 6:120–8. 24834258 PMC4017514

[14] Robinson MD Mccarthy DJ Smyth GK. edgeR: a Bioconductor Package for Differential Expression Analysis of Digital Gene Expression Data. Bioinformatics (2010) 26:139–40. 10.1093/bioinformatics/btp616 19910308 PMC2796818

[15] Robin X Turck N Hainard A. pROC: an Open-Source Package for R and S+ to Analyze and Compare ROC Curves. BMC Bioinformatics (2011) 12:77. 10.1186/1471-2105-12-77 21414208 PMC3068975

[16] Sing T Sander O Beerenwinkel N Lengauer T. ROCR: Visualizing Classifier Performance in R. Bioinformatics (2005) 21:3940–1. 10.1093/bioinformatics/bti623 16096348

[17] You JS Jones PA. Cancer Genetics and Epigenetics: Two Sides of the Same coin? Cancer Cell (2012) 22:9–20. 10.1016/j.ccr.2012.06.008 22789535 PMC3396881

[18] Jones PA Issa J-PJ Baylin S. Targeting the Cancer Epigenome for Therapy. Nat Rev Genet (2016) 17:630–41. 10.1038/nrg.2016.93 27629931

[19] Okugawa Y Grady WM Goel A. Epigenetic Alterations in Colorectal Cancer: Emerging Biomarkers. Gastroenterology (2015) 149:1204–25. 10.1053/j.gastro.2015.07.011 26216839 PMC4589488

[20] Kong X Chen J Xie W Brown SM Cai Y Wu K Defining UHRF1 Domains that Support Maintenance of Human Colon Cancer DNA Methylation and Oncogenic Properties. Cancer Cell (2019) 35:633–48. 10.1016/j.ccell.2019.03.003 30956060 PMC6521721

[21] Sugimachi K Matsumura T Shimamura T Hirata H Uchi R Ueda M Aberrant Methylation of FOXE1 Contributes to a Poor Prognosis for Patients with Colorectal Cancer. Ann Surg Oncol (2016) 23:3948–55. 10.1245/s10434-016-5289-x 27271927

[22] Kandimalla R Linnekamp JF Van Hooff S Castells A Llor X Andreu M Methylation of WNT Target Genes AXIN2 and DKK1 as Robust Biomarkers for Recurrence Prediction in Stage II colon Cancer. Oncogenesis (2017) 6:e308. 10.1038/oncsis.2017.9 28368388 PMC5520503

[23] Tse JWT Jenkins LJ Chionh F Mariadason JM. Aberrant DNA Methylation in Colorectal Cancer: What Should We Target? Trends Cancer (2017) 3:698–712. 10.1016/j.trecan.2017.08.003 28958388

[24] Takatsu H Hase K Ohmae M Ohshima S Hashimoto K Taniura N CD300 Antigen like Family Member G: A Novel Ig Receptor like Protein Exclusively Expressed on Capillary Endothelium. Biochem Biophysical Res Commun (2006) 348:183–91. 10.1016/j.bbrc.2006.07.047 16876123

[25] Umemoto E Takeda A Jin S. Dynamic Changes in Endothelial Cell Adhesion Molecule nepmucin/CD300LG Expression under Physiological and Pathological Conditions. PLoS One (2013) 8:e83681. 10.1371/journal.pone.0083681 24376728 PMC3871519

[26] Miles FL Pruitt FL Van Golen KL Cooper CR. Stepping Out of the Flow: Capillary Extravasation in Cancer Metastasis. Clin Exp Metastasis (2008) 25:305–24. 10.1007/s10585-007-9098-2 17906932

[27] Taguchi T. Mannosyl (Alpha-1,3[6?]-)-Glycoprotein Beta-1,4-N-Acetylglucosaminyltransferase, Isozyme C (Putative) (MGAT4C). In: Handbook of Glycosyltransferases and Related Genes (2014) p. 257–63. 10.1007/978-4-431-54240-7_134

[28] Demichelis F Setlur SR Banerjee S Chakravarty D Chen JYH Chen CX Identification of Functionally Active, Low Frequency Copy Number Variants at 15q21.3 and 12q21.31 Associated with Prostate Cancer Risk. Proc Natl Acad Sci (2012) 109:6686–91. 10.1073/pnas.1117405109 22496589 PMC3340033

