# Supplementary figures and images for: Integrative Analysis of DNA Methylation and Gene Expression Profiles Identifies Colorectal Cancer-Related Diagnostic Biomarkers
Source: Pathol Oncol Res. 2021 Jul 21;27:1609784. doi: 10.3389/pore.2021.1609784 (PMC8333028; doi:10.3389/pore.2021.1609784)

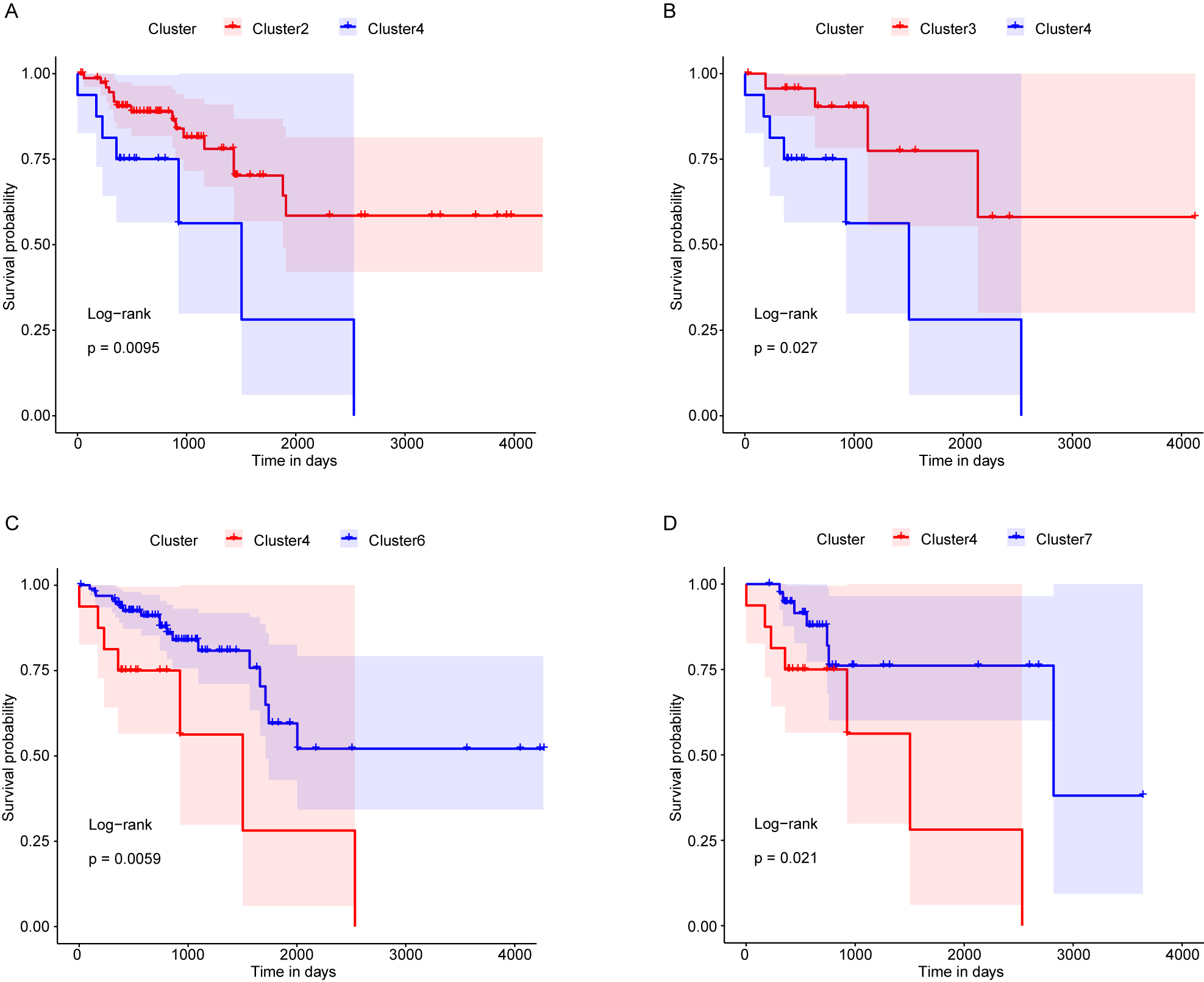

Supplement: Supplementary file 1 [file Image1.TIF]
